# Associations between type 2 diabetes mellitus and chronic liver diseases: evidence from a Mendelian randomization study in Europeans and East Asians

**DOI:** 10.3389/fendo.2024.1338465

**Published:** 2024-03-01

**Authors:** Yue Zhao, Di Li, Hanyu Shi, Wei Liu, Jiaojiao Qiao, Shanfu Wang, Yiwei Geng, Ruiying Liu, Feng Han, Jia Li, Wei Li, Fengyun Wu

**Affiliations:** ^1^ Department of Surgery, Hospital of the First Mobile Corps of the Chinese People’s Armed Police Force, Dingzhou, Hebei, China; ^2^ Department of Internal Medicine, Hospital of the First Mobile Corps of the Chinese People’s Armed Police Force, Dingzhou, Hebei, China; ^3^ Department of General Surgery, Shandong Corps Hospital of Chinese People’s Armed Police Force, Jinan, China; ^4^ Department of Nursing, Hospital of the First Mobile Corps of the Chinese People’s Armed Police Force, Dingzhou, Hebei, China; ^5^ School of Statistic and Data Science, Jiangxi University of Finance and Economics, Nanchang, Jiangxi, China; ^6^ Department of Health and Epidemic Prevention, Hospital of the First Mobile Corps of the Chinese People’s Armed Police Force, Dingzhou, Hebei, China; ^7^ Department of General Surgery, The 980Hospital of the Chinese People's Liberation Army (PLA) Joint Logistics Support Force (Primary Bethune International Peace Hospital of Chinese People's Liberation Army (PLA), Shijiazhuang, Hebei, China; ^8^ Department of General Surgery, Characteristic Medical Center of the Chinese People’s Armed Police Force, Tianjin, China

**Keywords:** Mendelian randomization, type 2 diabetes mellitus, nonalcoholic fatty liver disease, hepatocellular carcinoma, viral hepatitis, hepatitis B virus infection, hepatitis C virus infection

## Abstract

**Objective:**

Multiple observational studies have demonstrated an association between type 2 diabetes mellitus (T2DM) and chronic liver diseases (CLDs). However, the causality of T2DM on CLDs remained unknown in various ethnic groups.

**Methods:**

We obtained instrumental variables for T2DM and conducted a two-sample mendelian randomization (MR) study to examine the causal effect on nonalcoholic fatty liver disease (NAFLD), hepatocellular carcinoma (HCC), viral hepatitis, hepatitis B virus (HBV) infection, and hepatitis C virus (HCV) infection risk in Europeans and East Asians. The primary analysis utilized the inverse variance weighting (IVW) technique to evaluate the causal relationship between T2DM and CLDs. In addition, we conducted a series of rigorous analyses to bolster the reliability of our MR results.

**Results:**

In Europeans, we found that genetic liability to T2DM has been linked with increased risk of NAFLD (IVW : OR =1.3654, 95% confidence interval [CI], 1.2250-1.5219, p=1.85e-8), viral hepatitis (IVW : OR =1.1173, 95%CI, 1.0271-1.2154, p=0.0098), and a suggestive positive association between T2DM and HCC (IVW : OR=1.2671, 95%CI, 1.0471-1.5333, p=0.0150), HBV (IVW : OR=1.1908, 95% CI, 1.0368-1.3677, p=0.0134). No causal association between T2DM and HCV was discovered. Among East Asians, however, there was a significant inverse association between T2DM and the proxies of NAFLD (ALT: IVW OR=0.9752, 95%CI 0.9597-0.9909, p=0.0021; AST: IVW OR=0.9673, 95%CI, 0.9528-0.9821, p=1.67e-5), and HCV (IVW: OR=0.9289, 95%CI, 0.8852-0.9747, p=0.0027). Notably, no causal association was found between T2DM and HCC, viral hepatitis, or HBV.

**Conclusion:**

Our MR analysis revealed varying causal associations between T2DM and CLDs in East Asians and Europeans. Further research is required to investigate the potential mechanisms in various ethnic groups, which could yield new insights into early screening and prevention strategies for CLDs in T2DM patients.

## 1 Introduction

Diabetes mellitus, an enormous public health concern, imposes staggering health and financial burdens worldwide (1, 2). There are two primary types of diabetes: Type 1 Diabetes Mellitus (T1DM) and Type 2 Diabetes Mellitus (T2DM) (3). Approximately 10% of adults worldwide suffer from T2DM, making it the most prevalent form of diabetes (4).T2DM, characterized by hyperglycemia resulting from disturbed glycemic homeostasis (5), is precipitated by defective insulin secretory responses and action. The liver, which is essential to glucose homeostasis by supplying endogenous sugar, is the first organ to receive insulin following its release from pancreatic islets (6). Moreover, hepatic glucose metabolic disorders could contribute to fast hyperglycemia in diabetic patients (7, 8).

Considering the important role of the liver in the pathogenesis and treatment of diabetes, various liver diseases have been associated with T2DM (9, 10). Previous studies have demonstrated a connection between T2DM and chronic liver diseases (CLDs), such as Non-alcoholic fatty liver disease (NAFLD), hepatocellular carcinoma (HCC) and viral hepatitis (11–13). A large meta-analysis from 156 studies estimated that the global prevalence of NAFLD among patients with T2DM was 65.04% (14). It was reported that coexistence of NAFLD and T2DM is common in everyday outpatient practice (15). There is a bidirectional relationship between NAFLD and T2DM that is confirmed by epidemiological data, clinical picture, diagnosis and pathomechanisms (16). However, the complex causal link between NAFLD and T2DM is still controversial, and it is important to bring the attention of clinicians and researchers to the relationship between these two metabolic diseases in order to prevent adverse clinical outcomes (16). Viral hepatitisis an acute or chronic inflammation of the liver parenchyma caused by viruses. There are 5 common types of viral hepatitis: A, B, C, D, and E, and most deaths from viral hepatitis are due to hepatitis B and hepatitis C (17). It is worth noting that hepatitis B Virus (HBV) and hepatitis C Virus (HCV) infections were reportedly higher among T2DM patients (18). However, other studies reported that the risk of HCV infection was rather low in patients with T2DM (19, 20). The discrepancies may be attributable to the sample sizes, target populations, control sources, potential biases from residual confounding, and reverse causation of the respective studies. These highlight how crucial it is to manage T2DM in CLDs patients and to make a firm determination of the causal relationships between T2DM and CLDs.

Mendelian Randomization (MR), a developing epidemiological technique, examines the effect of genotypic variation (exposure) on a phenotype (outcome) from a genetic standpoint. Using germline genetic variance as instrumental variables (IVs), MR analysis assesses causation between exposure and outcomes, thereby avoiding biases caused by confounding variables or reverse causation (21). Previous MR research assessed the relationship between NAFLD and T2DM in Europeans but skipped East Asians (22). To comprehensively evaluate the role of T2DM in CLDs across both demographics, we conducted a comprehensive two-sample MR study.

## 2 Materials and methods

### 2.1 Study design

**Figure 1:**
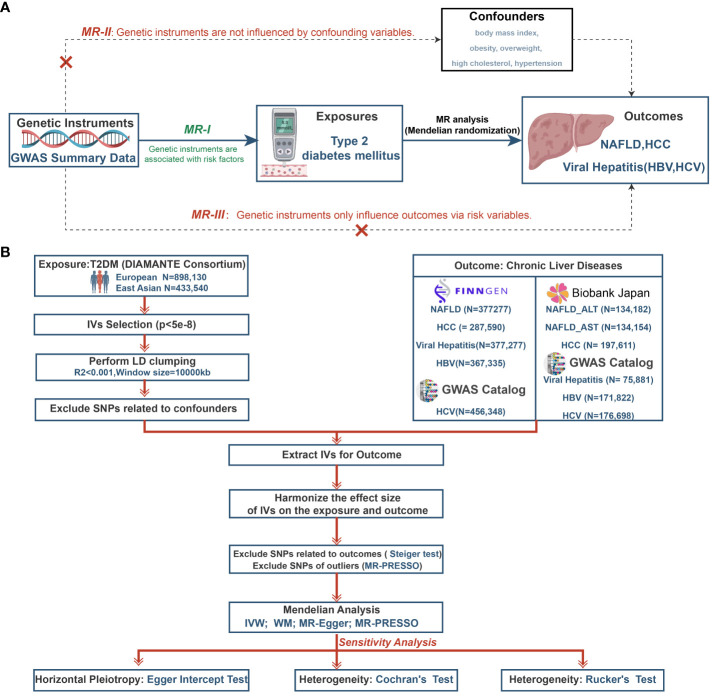
The study design of MR analysis **(A)** and the overall workflow **(B)**. MR, mendelian randomization; GWAS, genome-wide association study; NAFLD, non-alcoholic fatty liver disease; HCC, hepatocellular carcinoma; HBV, hepatitis B virus; HCV, hepatitis C virus; T2DM, type 2 diabetes mellitus; DIAMANTE, Diabetes Meta-analysis of Trans-ethnic Association Studies; IVs, instrumental variables; LD, linkage disequilibrium; SNPs, single-nucleotide polymorphisms; ALT, Alanine aminotransferase; AST, Aspartate aminotransferase; MR-PRESSO, Mendelian randomization pleiotropy residual sum and outlier; IVW, inverse-variance weighted; WM, weighted median.

A two-sample MR study was designed to investigate the causative role of T2DM on the risk of five CLDs (NAFLD, HCC, viral hepatitis, HBV and HCV). Three critical assumptions for MR analysis must be satisfied (see **Figure 1A**) (1): IVs should be associated with exposure factors; (2) IVs should not be associated with confounding factors; and (3) IVs should only affect the outcome through the exposure (23). The study relied on publicly available genome-wide association studies (GWAS) data. We estimated the relationships between T2DM and CLDs in Europeans and East Asians and **Figure 1B** depicts the study’s overall workflow.

### 2.2 Data sources

#### 2.2.1 Genetic association datasets for T2DM

The GWAS summary statistics for T2DM were obtained from the Diabetes Meta-analysis of Trans-ethnic Association Studies (DIAMANTE) Consortium, including 898,130 European individuals (n=74,124 case patients and 824,006 control participants) and 433,540 East Asian individuals(n=77,418 case patients and 356,122 control participants) (24).

#### 2.2.2 Genetic association datasets for CLDs

Summary statistics from the GWAS of European populations for four outcomes were procured from the FinnGen consortium, which is collecting genetic data based on FinnGen registries. For this study, we utilized data from their R9 release (https://r9.finngen.fi/). This release includes data regarding NAFLD (2,275 cases and 375,002 controls), HCC (453 cases and 287,137 controls), viral hepatitis (2,143 cases and 375,134 controls), and HBV (885 cases and 366,450 controls). The HCV summary statistics (219 cases and 456,129 controls) were obtained from Jiang’s research (25) and were also available in the GWAS catalog (https://www.ebi.ac.uk/gwas/).

In the absence of valid GWAS for NAFLD in East Asians, we used Alanine aminotransferase (ALT) and Aspartate aminotransferase (AST) levels from the Biobank Japan GWAS as surrogates for NAFLD, following a precedent established in a previous MR study (26). We eventually obtained summary statistics for East Asian for NAFLD (ALT: n=134,182, AST: n=134,154), and HCC (1,866 cases and 195,745 controls) from the IEU OPEN GWAS PROJECT (https://gwas.mrcieu.ac.uk/). The remaining summary statistics for viral hepatitis (117 cases and 75,764 controls), HBV (2,234 cases and 169,588 controls), and HCV (7,110 cases and 169,588 controls) were obtained from studies conducted by Walters RG and Sakaue S (27, 28). **Table 1** provides a comprehensive summary of the data sources used for each exposure and outcome.

**Table 1:** Profiles of exposure and outcomes in GWAS datasets.

Trait	GWAS ID (Data sources)	Data Type	Case	Control	Ethnicity	Consortium
T2DM	PMID: 35551307	Exposure	74,124	824,006	European	DIAMANTE
T2DM	PMID: 35551307	Exposure	77,418	356,122	East Asian	DIAMANTE
NAFLD	finngen_R9_NAFLD	Outcome	2,275	375,002	European	FinnGen biobank
HCC	finngen_R9_C3_HEPATOCELLU_CARC_EXALLC	Outcome	453	287,137	European	FinnGen biobank
Viral Hepatitis	finngen_R9_AB1_VIRAL_HEPATITIS	Outcome	2,143	375,134	European	FinnGen biobank
HBV	finngen_R9_K11_CHRONHEP	Outcome	885	366,450	European	FinnGen biobank
HCV	GCST90041714(PMID: 34737426)	Outcome	219	456,129	European	GWAS catalog
ALT	bbj-a-6(PMID: 29403010)	Outcome	134,182		East Asian	Biobank Japan
AST	bbj-a-8(PMID: 29403010)	Outcome	134,154		East Asian	Biobank Japan
HCC	bbj-a-158(PMID: 32514122)	Outcome	1,866	195,745	East Asian	Biobank Japan
Viral Hepatitis	GCST90246018(PMID: 37601966)	Outcome	117	75,764	East Asian	GWAS catalog
HBV	GCST90018584(PMID: 34594039)	Outcome	2,234	169,588	East Asian	GWAS catalog
HCV	GCST90018585(PMID: 34594039)	Outcome	7,110	169,588	East Asian	GWAS catalog

GWAS, genome-wide association study; ID, identity; T2DM, type 2 diabetes mellitus; DIAMANTE, Diabetes Meta-analysis of Trans-ethnic Association Studies; NAFLD, non-alcoholic fatty liver disease; HCC, hepatocellular carcinoma; HBV, hepatitis B virus; HCV, hepatitis C virus; ALT, Alanine aminotransferase; AST, Aspartate aminotransferase.

### 2.3 Genetic instrument selection and evaluation

The single-nucleotide polymorphisms (SNPs) associated with T2DM were selected based on the following criteria (1): The phenotypes should be significantly related with IVs (P < 5 × 10^-8^). (2) The related linkage disequilibrium (LD) of r^2^<0.001 and clumping with a 10000kb window were considered. (3) IVs should have at least five variants as biallelic SNPs (29).

Variance (R2) and the F-statistic were utilized to evaluate the robustness of IVs in order to avoid tool bias. F-statistic was computed for each SNP using the following formula: F = (beta/se)^2^ (30). To reduce bias caused by weak IVs, an F-statistic greater than 10 was considered significant for the IV-exposure association (31). Statistical power was calculated for each outcome using the online tool (https://shiny.cnsgenomics.com/mRnd/) (32). A sufficient strength of over 80% was advised. We also searched all eligible SNPs with PhenoScanner V2(http://www.phenoscanner.medschl.cam.ac.uk/) to exclude SNPs associated with potential confounders including body mass index, obesity, overweight, high cholesterol, hypertension, and alcohol consumption (33–35). In addition, we harmonized the summary statistics and removed SNPs with palindromic sequences.

### 2.4 Statistical analysis

As the primary method, the random-effect inverse-variance weighted (IVW) approach was utilized. Three additional MR methods, the weighted median (WM), MR-Egger, and Mendelian randomization pleiotropy residual sum and outlier (MR-PRESSO), were also employed in this study. The IVW method is an extension of the Wald ratio estimator based on meta-analytic principles that can generate relatively precise estimates that are not influenced by horizontal pleiotropy (36). MR-Egger and WM could provide more stable estimates and correct potential pleiotropy in a broader set of scenarios but are less efficient (37, 38). The MR-PRESSO was implemented to identify and remove pleiotropic outliers so that the causal impact estimate is obtained (39). Considering the multiple comparisons, the Bonferroni method was performed to rectify overall type I errors, and p<0.01 (0.05/5) was considered statistically significant association, while p<0.05 was considered suggestive association (22, 40).

Multiple sensitivity analyses were implemented to guarantee the uniformity and reliability of the MR results. Cochran’s and Rucker’s Q test were used to detect heterogeneity, where P<0.05 indicated the presence of heterogeneity (41). Using the MR-Egger intercept test, we then evaluated the directional horizontal pleiotropy; p>0.05 indicated that there was no directional horizontal pleiotropy (42). Finally, leave one-out analysis was performed to evaluate whether the MR estimate was driven or biased by a single SNP (43). To explore the possible reverse causality, we performed the Steiger filtering test to evaluate the direction of causality for each IV on exposure and outcome. When their directions are “FALSE”, related SNPs were removed and the MR analysis would be reassessed (44). A two-sided p-value < 0.05 was considered statistically significant. All statistical analyses were performed in R software 4.3.1 (R Foundation for Statistical Computing) using R packages “Two Sample MR”.

## 3 Results

### 3.1 Selection of IVs

A total of 44 SNPs associated with potential confounders of NAFLD (body mass index, obesity, overweight, high cholesterol, and hypertension) were removed, and 131 SNPs were selected for analysis in Europeans. In East Asians, 17 and 17 SNPs associated with possible confounders of the ALT and AST were eliminated. Through the application of MR-PRESSO, outlier SNPs (rs79747549) linked with ALT were dropped. In the end, 65 and 66 SNPs corresponding to ALT and AST were chosen from East Asians as candidates (**Supplementary Table S1**). Regarding the SNPs associated with HCC, three SNPs, namely rs429358, rs28663084, and rs1260326, were excluded from the analysis based on the results of MR-PRESSO and the Steiger test, as well as their association with potential confounding variables. Ultimately, a set of 169 SNPs were identified as IVs in the European population. In the study conducted on the HCC patients of East Asians, two SNPs, namely rs9948462 and rs1260326, were eliminated from the analysis depending on the MR-PRESSO and Steiger test, as well as their association with potential confounding variables. Eventually, a total of 83 SNPs were chosen for further investigation, as outlined in **Supplementary Table S2**. For viral hepatitis, a total of 173 and 85 SNPs were selected in Europeans and East Asians, respectively. Specifically, Europeans selected 173 SNPs for HBV and 178 SNPs for HCV. In comparison, East Asians selected 86 SNPs for HBV and 86 SNPs for HCV (**Supplementary Table S3**). **Supplementary Table S4** shows the detailed eliminated SNPs in the selection of IVs. It is worth noting that all selected IVs exhibited F-statistics greater than 10.

### 3.2 Causal effect of T2DM on NAFLD

**Figure 2:**
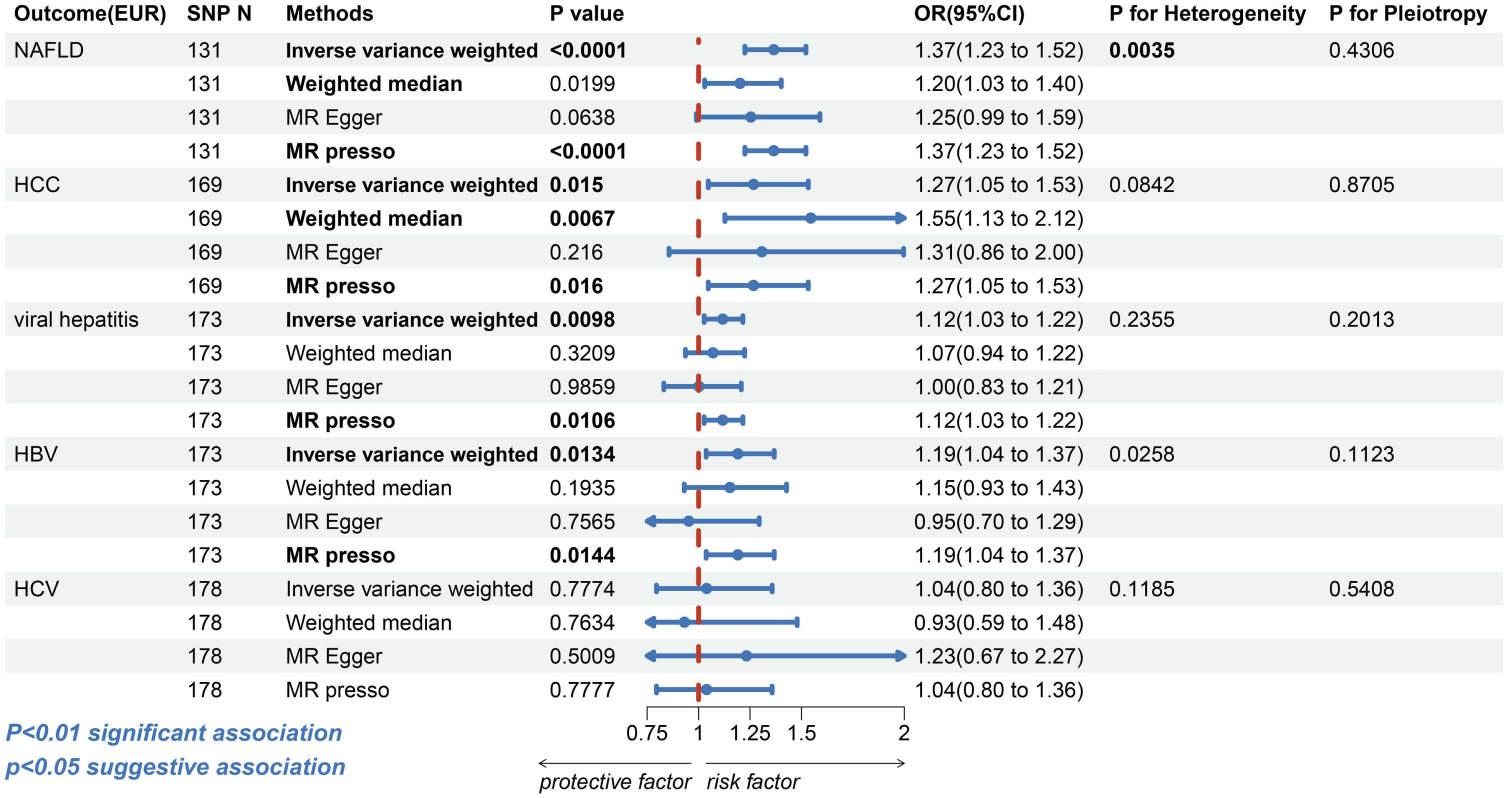
The forest plot depicting the genetic susceptibility to T2DM in Europeans with CLDs. T2DM, type 2 diabetes mellitus; CLDs, chronic liver diseases; EUR, Europeans; NAFLD, non-alcoholic fatty liver disease; HCC, hepatocellular carcinoma; HBV, hepatitis B virus; HCV, hepatitis C virus; SNP, single-nucleotide polymorphisms; IVW, inverse-variance weighted; WM, weighted median; MR-PRESSO, Mendelian randomization pleiotropy residual sum and outlier; OR, odds ratio; CI, confidence interval. Statistical significance was set at p< 0.05.

In Europeans, the risk of NAFLD was found to be higher among patients with T2DM (IVW: OR=1.3654, 95% confidence interval [CI] 1.2250-1.5219, p=1.85e-8; WM: OR=1.2013,95%CI, 1.0295-1.4019, p=0.0199; MR-Egger: OR=1.2540, 95%CI, 0.9891-1.5899, p=0.0638, MR-PRESSO: OR=1.3654, 95%CI, 1.2250-1.5219, p=1.08e-7, **Figure 2** and **Supplementary Figure S1**). Despite the presence of heterogeneity (Cochran’s Q test: p=0.0035 and Rucker’s Q test: p=0.0033, **Supplementary Table S5**), no significant outliers were identified, and the similar result from MR-PRESSO and leave-one-out analysis (**Supplementary Figure S1**) supports the consistency of the conclusion. Also, no directional horizontal pleiotropy was observed in the MR analysis (MR-Egger intercept=-0.0063, p=0.4306, **Supplementary Table S5**). Furthermore, the MR Steiger test yielded no indication of reverse causality.

**Figure 3:**
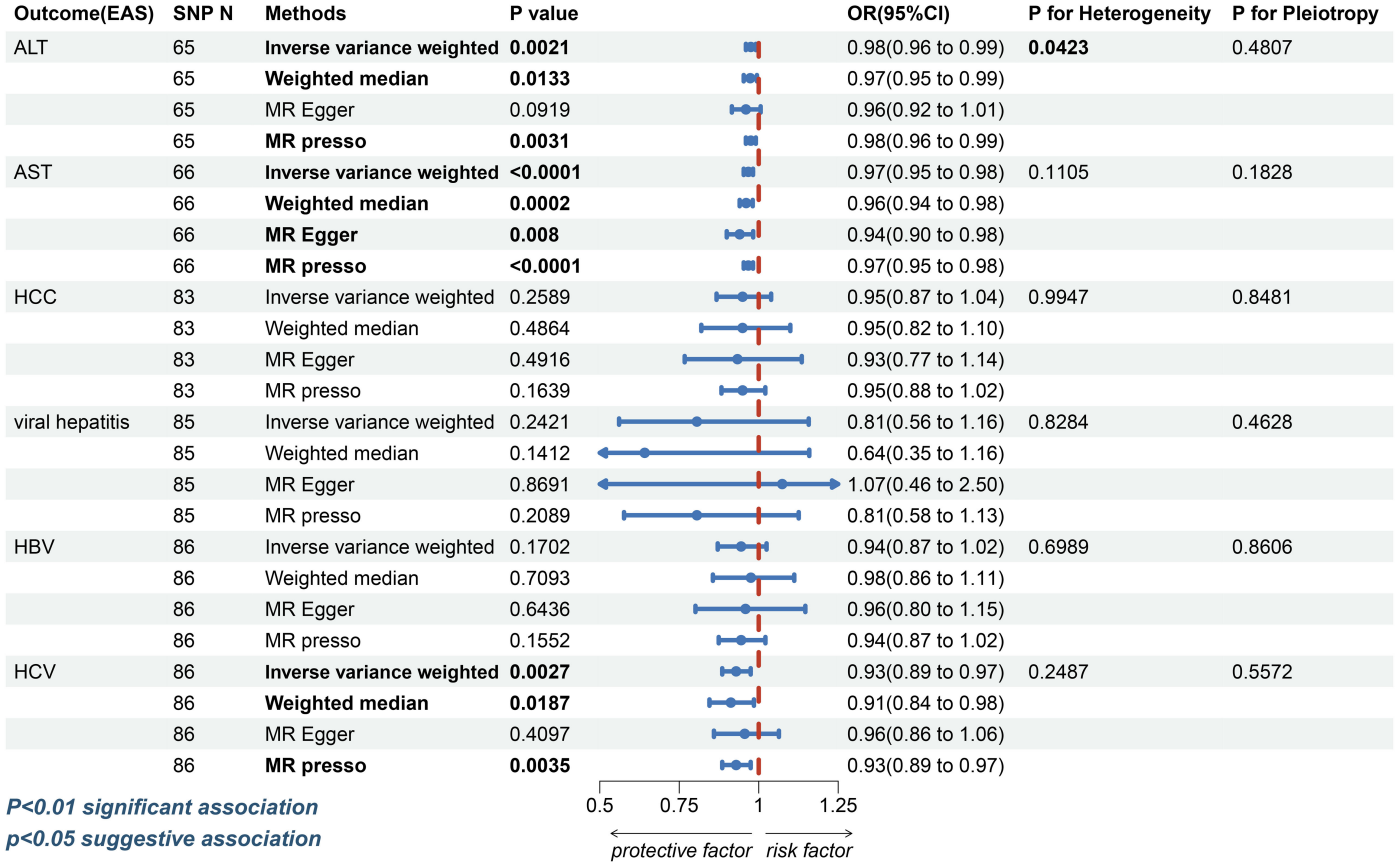
The forest plot depicting the genetic susceptibility to T2DM in East Asians with CLDs. T2DM, type 2 diabetes mellitus; CLDs, chronic liver diseases; EAS, East Asians; ALT, Alanine aminotransferase; AST, Aspartate aminotransferase; HCC, hepatocellular carcinoma; HBV, hepatitis B virus; HCV, hepatitis C virus; SNP, single-nucleotide polymorphisms; IVW, inverse-variance weighted; WM, weighted median; MR-PRESSO, Mendelian randomization pleiotropy residual sum and outlier; OR, odds ratio; CI, confidence interval. Statistical significance was set at p< 0.05.

In East Asians, however, the risk of elevated ALT (IVW: OR=0.9752, 95%CI, 0.9597-0.9909, p=0.0021; WM: OR=0.9734, 95%CI, 0.9528–0.9944, p=0.0133; MR-Egger: OR=0.9597, 95% CI, 0.9155-1.0060, p=0.0919; MR-PRESSO: OR=0.9752, 95%CI, 0.9597-0.9909, p=0.0031, **Figure 3** and **Supplementary Figure S2**) and AST (IVW: OR=0.9673, 95%CI, 0.9528-0.9821, p=1.67e-5; WM: OR=0.9604, 95%CI, 0.9401-0.9811, p=0.0002; MR-Egger: OR=0.9401, 95%CI, 0.8994-0.9826, p=0.0080; MR-PRESSO: OR=0.9673, 95%CI, 0.9528-0.9821, p=5.74e-5; **Figure 3** and **Supplementary Figure S3**) was found to decrease in patients with T2DM. In the sensitivity analysis, there was no evidence of horizontal directional pleiotropy (ALT: MR-Egger intercept=0.0013, p=0.4807; AST: MR-Egger intercept=0.0024, p=0.1828, **Supplementary Table S5**). Despite the ALT index exhibited heterogeneity (ALT: Cochran’s Q test: p=0.0423 and Rucker’s Q test: p=0.0392; AST: Cochran’s Q test: p=0.1105 and Rucker’s Q test: p=0.1270, **Supplementary Table S5**), the leave-one-out analysis (**Supplementary Figure S3**) validate the reliability of the conclusion in Asian inhabitants.

### 3.3 Causal effect of T2DM on HCC

For HCC, a possible causal relationship was observed between T2DM and HCC in European populations (IVW: OR=1.2671, 95%CI, 1.0471-1.5333, p=0.0150; WM: OR=1.5455, 95%CI 1.1280-2.1175, p=0.0067; MR-Egger: OR=1.3076, 95%CI 0.8563-1.9965, p=0.2160; MR-PRESSO: OR=1.2671, 95%CI 1.0471-1.5333, p=0.0160, **Figure 2** and **Supplementary Figure S4**). However, the association between T2DM and HCC (IVW: OR=0.9493, 95%CI, 0.8673-1.0391, p=0.2589; WM: OR=0.9491, 95% CI 0.8194-1.0094, p=0.4864; MR-Egger: OR=0.9332, 95%CI 0.7671-1.1354, p=0.4916; MR-PRESSO: OR=0.9493, 95%CI, 0.8828-1.0208, p=0.1639, **Figure 3** and **Supplementary Figure S5**) was insignificant among East Asian individuals. Neither heterogeneity nor horizontal pleiotropy was seen across any humanity (**Supplementary Table S5**).

### 3.4 Causal effect of T2DM on viral hepatitis

Next, we discovered that patients with T2DM in European populations had an increased risk of contracting viral hepatitis (IVW: OR=1.1173, 95%CI, 1.0271-1.2154, p=0.0098; WM: OR=1.0703, 95%CI, 0.9360-1.2239, p=0.3209; MR-Egger: OR=1.0017, 95%CI, 0.8310-1.2075, p=0.9859; MR-PRESSO: OR=1.1173, 95% CI, 1.0271-1.2154, p=0.0106, **Figure 2** and **Supplementary Figure S6**). More specifically, T2DM was possibly linked with HBV (IVW: OR=1.1908, 95%CI, 1.0368-1.3677, p=0.0134; WM: OR=1.1525, 95%CI, 0.9305-1.4274, p=0.1935; MR-Egger: OR=0.9526, 95%CI, 0.7009-1.2946, p=0.7565; MR-PRESSO: OR=1.1908, 95%CI, 1.0368-1.3677, p=0.0144, **Figure 2** and **Supplementary Figure S7**). The association between T2DM and HCV was insignificant (IVW: OR=1.0393, 95%CI, 0.7956-1.3575, p=0.7774; WM: OR=0.9314, 95%CI, 0.5866-1.4791, p=0.7634; MR-Egger: OR=1.2330, 95%CI, 0.6708-2.2664, p=0.5009; MR-PRESSO: OR=1.0393, 95%CI, 0.7956-1.3575, p=0.7777, **Figure 2** and **Supplementary Figure S8**). There was no evidence of horizontal directional pleiotropy in any of these MR analyses (**Supplementary Table S5**). Notwithstanding, the analysis with HBV revealed heterogeneity (Cochran’s Q test: p=0.0258, Rucker’s Q test: p=0.0319); however, the leave-one-out analysis validate the conclusion’s validity (**Supplementary Figure S7**).

In East Asians, the causal relationship between T2DM and viral hepatitis, HBV, was considered invalid by four different methods (**Figure 3** and **Supplementary Figures S9, 10**). Nevertheless, it was discovered that patients with T2DM had a lower risk of contracting HCV (IVW: OR=0.9289, 95%CI, 0.8852- 0.9747, p=0.0027; WM: OR=0.9123, 95%CI, 0.8450-0.9849, p=0.0187; MR-Egger: OR=0.9559, 95%CI, 0.8591-1.0635, p=0.4097; MR-PRESSO: OR=0.9289, 95%CI, 0.8852-0.9747, p=0.0035, **Figure 3** and **Supplementary Figure S11**). Horizontal pleiotropy and heterogeneity were not observed in any of the three diseases (**Supplementary Table S5**).

## 4 Discussion

**Figure 4:**
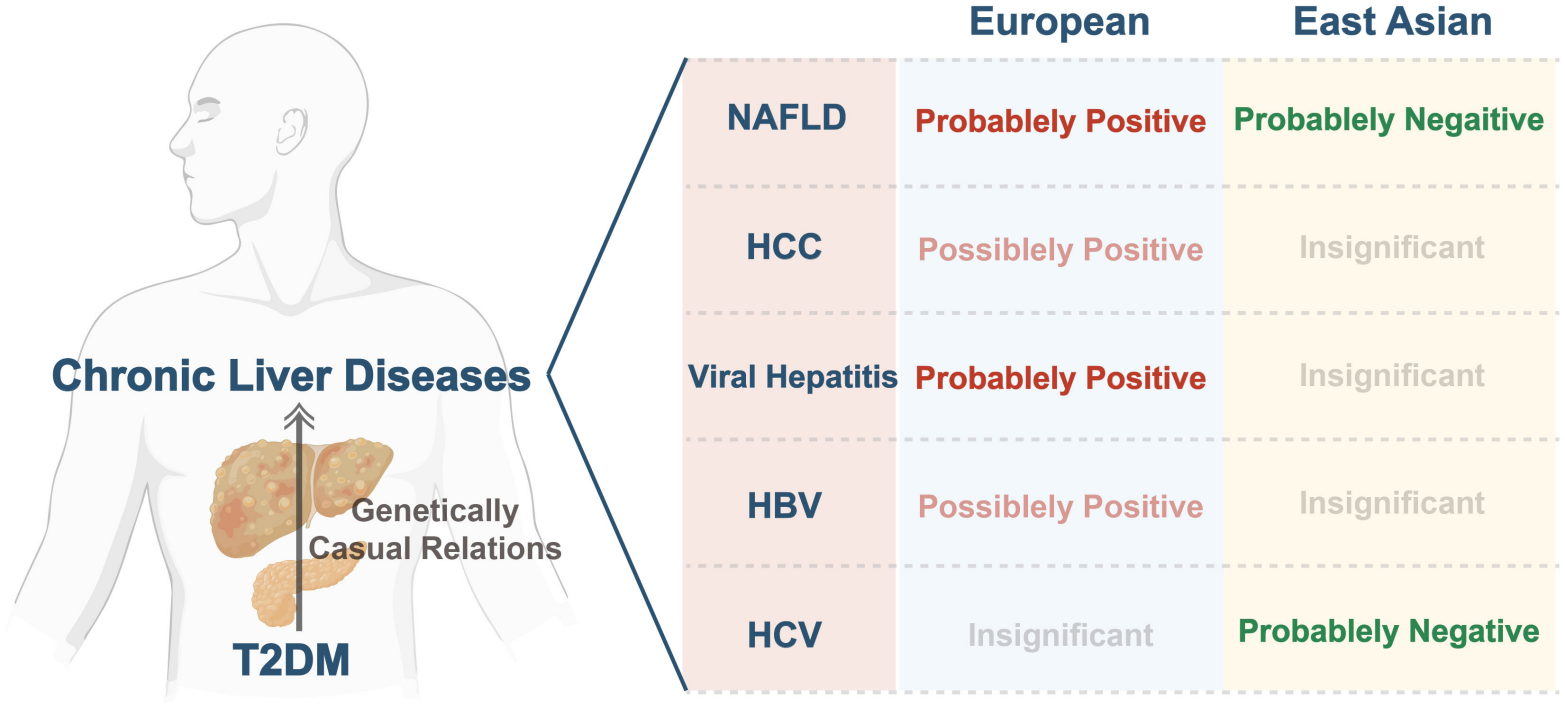
Summary of genetically causal relations between T2DM and CLDs across various ethnic backgrounds. T2DM, type 2 diabetes mellitus; CLDs, chronic liver diseases; NAFLD, non-alcoholic fatty liver disease; HCC, hepatocellular carcinoma; HBV, hepatitis B virus; HCV, hepatitis C virus.

To the extent of our knowledge, this is the first study leveraging MR to comprehensively investigate the association of genetic predictors of T2DM with CLDs across various ethnic backgrounds. Our analysis indicates that T2DM was probably linked with NAFLD and viral hepatitis, and possibly associated with HCC, and HBV in Europeans. In East Asians, our findings suggest a significant inverse correlation between T2DM and ALT, AST, and HCV. However, we did not find any causal association between T2DM and HCC, viral hepatitis, and HBV. **Figure 4**.

Epidemiological data has revealed that the age-adjusted relative risk of NAFLD is approximately 5.36 times greater in individuals with T2DM as opposed to the general non-diabetic population (45). Prior MR studies also presented evidence supporting correlations between genetic predisposition to T2DM and an escalated risk of NAFLD, particularly within European populations (46). These findings align with our MR analysis, albeit our study was conducted on a larger sample size. Numerous potential mechanisms may underpin the associations such as insulin resistance (IR), altered lipid metabolism, and inflammation (47). T2DM is classified as a chronic low-grade inflammatory disease characterized by the elevated levels of interleukin-6 and tumor necrosis factor (TNF)-α (48, 49). By stimulating cellular kinase and inhibiting kappa B kinase, these inflammatory cytokines can induce IR (50). IR may deliver substrates and precursors (e.g., free fatty acids, glucose, and glycerlol) for *de novo* lipogenesis and mitochondrial β-oxidation, processes that induce hepatic steatosis and increase the susceptibility to NAFLD (47, 51, 52). Moreover, higher blood glucose levels can activate carbohydrate response element-binding protein (ChREBP) signaling pathway, which stimulates the expression of several glycolytic genes, and ChREBP overexpression induced stearoyl-CoA desaturase 1 expression, increasing liver fat content (50, 53, 54).

NAFLD patients usually have abnormal liver function (55). Previous studies have shown that ALT, AST and AST/ALT ratio can predict NAFLD, and AST/ALT are closely related to IR and T2DM (55, 56). High circulating ALT and AST are widely used proxies of NAFLD, although they are not specific markers of NAFLD (57). Interestingly, our MR studies showed there was an inverse association between T2DM and NAFLD Proxies (ALT, AST) in East Asians. A retrospective investigation conducted in coastal Eastern India revealed that NAFLD patients of Indian descent exhibited a lower body mass index (BMI) and a lower prevalence of diabetes when compared to those in Western populations (58). Hence, we formulated the hypothesis that, with the exception of genetic predisposition and environmental exposures, the lifestyle and body weight in the West may account for a portion of these variations among populations and ethnicities but it remains to be validated.

Regarding the relation between T2DM with HCC, a recent meta-analysis reported that T2DM is associated with a significantly higher risk of HCC (12). Another larger cohort study also showed that the incidence of HCC was three times higher than the general population compared to patients with T2DM (59). Several factors could explain this observation, firstly, long-term T2DM can cause oxidative stress and telomere shortening, which induces DNA damage, apoptosis and chromosomal instability in hepatocytes, increasing the risk of HCC (60, 61). Secondly, IR is highly suspected of being a cancerogenic condition, which may attribute to hyperinsulinemia and increased bioavailable insulin-like growth factor I (IGF-I) (59, 62). In Europeans, our MR study identified a causal effect of T2DM on HCC that was only suggestive; in East Asians, no causal effect was observed. The potential explanation for this association specific to ethnicity is still unknown. The observed discrepancies in outcomes across ethnic groups may potentially be attributed to variations in residual confounding and selection bias, factors that are unavoidable (26, 63). Additionally, environmental, genetic, dietary, and lifestyle factors may account for the inverse result. Thus, additional research is required to examine the ethnic variations in disease risk profiles in order to develop more effective treatment approaches.

Additionally, numerous studies have investigated the correlation between T2DM and viral hepatitis, such as HBV and HCV. HBV infection is the most prevalent chronic viral infection on a global scale, affecting roughly 30% of the world’s population in their lives; over 350 million individuals are chronic carriers of the virus (64). Also, it is estimated that around 71.1 million people are chronically infected with HCV, and that 1.75 million new cases of HCV infection were identified in 2015 (65). Previous study shows that patients with T2DM are more likely to be infected with HBV and HCV (18). This could be attributed to a multitude of possible factors. Firstly, HCV replication may be favored by hyperinsulinemia and/or the increased serum levels of free fatty acids observed in patients with T2DM (13). Then, T2DM has been associated with an immunocompromised condition, resulting in disruptions of immune function that potentially heighten the vulnerability to infection with HBV and HCV (66, 67). Also, it is worth noting that the process may be significantly influenced by interleukin-6, tumor necrosis factor-alpha, and various other immune-mediated pathways (9, 66, 68). Within the European population, this MR study revealed a suggestive association between T2DM and HBV and a positive correlation between T2DM and viral hepatitis. On the other hand, we discovered an inverse relationship between T2DM and HCV and absent relation between T2DM and viral hepatitis, HBV in East Asians. Considering the divergent outcomes observed in Eurasian populations, we posit that the relationship between T2DM and CLDs may indeed exhibit ethnic variations. Certainly, we consider the need for further investigation into potential molecular mechanisms and pathways involved in these ethnic disparities. Future studies, particularly those conducted in diverse ethnic populations, should delve deeper into understanding the intricate interplay between T2DM and CLDs. Our MR study also has certain shortcomings. First, comparatively small numbers of cases, such as NAFLD and viral hepatitis in East Asians, may result in low precision and potentially false negative results, which could influence the different MR results in different ancestral origins. Nevertheless, we have implemented the most extensive database of these diseases available, and additional research encompassing larger case sizes and ethnic backgrounds is necessary to definitively ascertain this correlation. Next, because there were zero SNPs as IVs when we set the threshold at 5e-8 in the GWAS of CLDs, we were unable to perform reverse MR analysis to estimate associations between CLDs and T2DM. Furthermore, the implementation of summarized GWAS precludes the ability to perform subgroup analyses according to gender and age. To mitigate such biases, future studies may employ within-family genome-wide association studies (GWASs) (69).

## 5 Conclusion

In summary, this research employed MR analysis to deduce a causal relationship between T2DM and CLDs by utilizing GWAS data from various ethnic populations. Our results showed the positive association between T2DM and NAFLD, viral hepatitis, HCC and HBV infection but not with HCV infection in Europeans. In contrast, in East Asians, T2DM was negatively associated with NAFLD and HCV but was not associated with HCC, viral hepatitis or HBV. The result revealed varying causal associations between T2DM and CLDs in East Asians and Europeans. Further clinical trials should be conducted in individuals of various ancestral origins to investigate the interaction between T2DM and CLDs in order to generate novel concepts for early screening and prevention of CLDs in patients with T2DM, as suggested by these findings.

## Data availability statement

The original contributions presented in the study are included in the article/**Supplementary Material**. Further inquiries can be directed to the corresponding authors.

## Author contributions

YZ: Conceptualization, Formal Analysis, Writing – original draft, Writing – review & editing. DL: Data curation, Methodology, Software, Writing – original draft. HS: Data curation, Methodology, Writing – original draft. WLu: Data curation, Software, Writing – original draft. JQ: Formal Analysis, Writing – original draft. SW: Project administration, Resources, Writing – review & editing. YG: Methodology, Writing – original draft. RL: Project administration, Writing – review & editing. FH: Resources, Writing – review & editing. JL: Data curation, Formal Analysis, Writing – review & editing. WLi: Conceptualization, Project administration, Validation, Writing – review & editing. FW: Conceptualization, Project administration, Supervision, Validation, Writing – review & editing.
