# Supplementary material for: Associations between type 2 diabetes mellitus and chronic liver diseases: evidence from a Mendelian randomization study in Europeans and East Asians
Source: Front Endocrinol (Lausanne). 2024 Mar 1;15:1338465. doi: 10.3389/fendo.2024.1338465 (PMC10941029; doi:10.3389/fendo.2024.1338465)
Supplement: Supplementary file 1 [file Image_1.pdf]

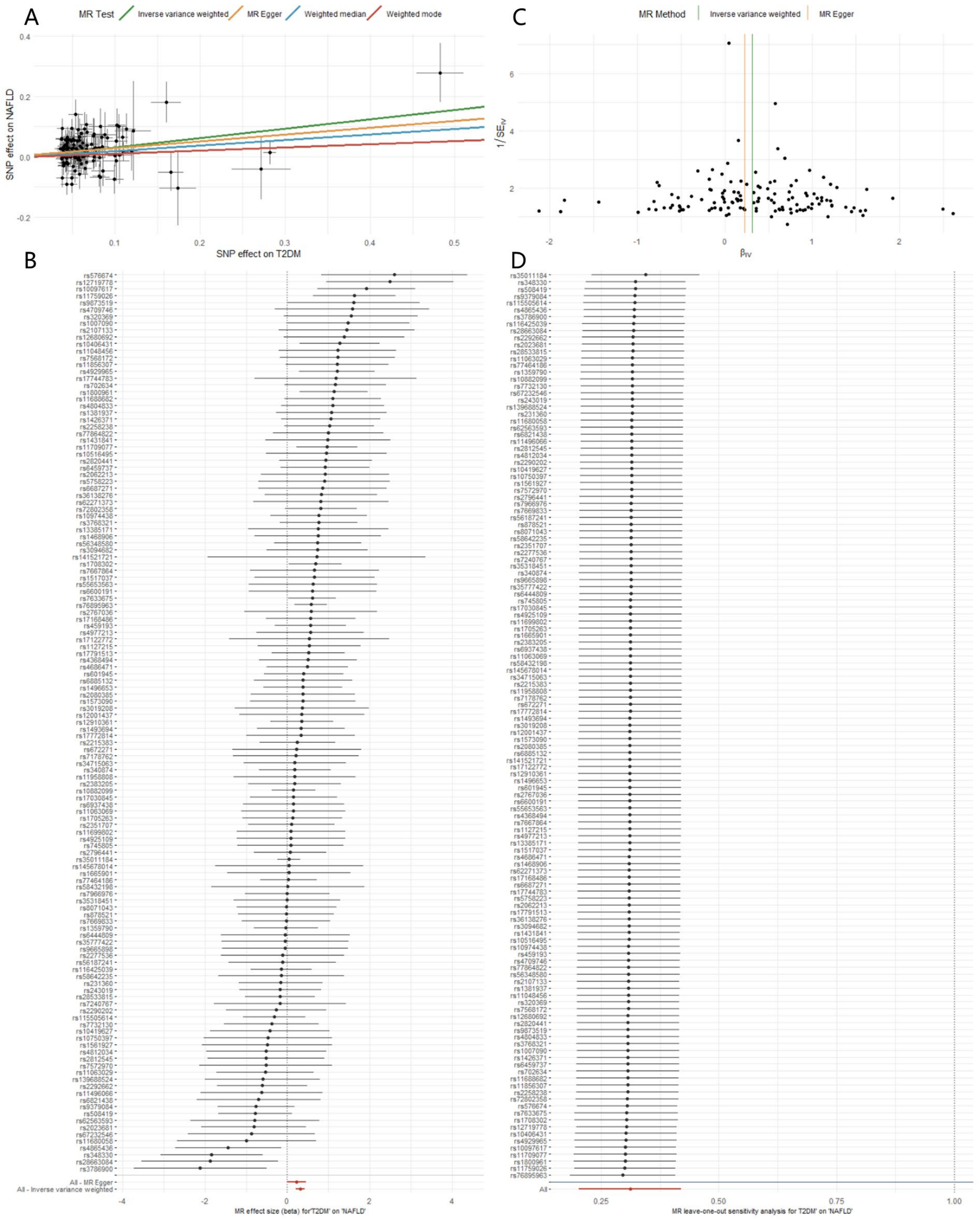

Figure S1 (A). Scatter plot referring to the effect sizes of the **SNP-T2DM association (x-axis, log OR) and the SNP-NAFLD in Europeans** associations (y-axis, log OR) with standard error bars. The slopes of the lines correspond to causal estimates using every of the four special methods (IVW random effects, MR-Egger, weighted median and weighted mode). (B). Forest plot shows the estimate of the impact of genetically increased T2DM risk on NAFLD risk in Europeans, where each black point represents the log OR for NAFLD per standard deviation extend in log OR for T2DM, and red points showing the combined causal estimate using all SNPs together in a single instrument, using every of the two different methods IVW and MR-Egger). Horizontal lines denote 95% confidence intervals (C). Funnel plot displaying the relationship

between the causal effect of T2DM on NAFLD in Europeans estimated using each individual SNP as a separate instrument against the inverse of the standard error of the causal estimate. Vertical lines exhibit the causal estimates using all SNPs blended into a single instrument for every of the two different methods (IVW random effects and MR-Egger). There is no tremendous asymmetry in the plot. (D). MR leave-one-out sensitivity analysis for T2DM on NAFLD in Europeans. Each black point represents the IVW MR method applied to estimate the causal impact of T2DM on NAFLD aside from that particular variant from the analysis. The red point depicts the IVW estimate using all SNPs. There are no instances where the exclusion of one unique SNP leads to dramatic adjustments in the standard result.

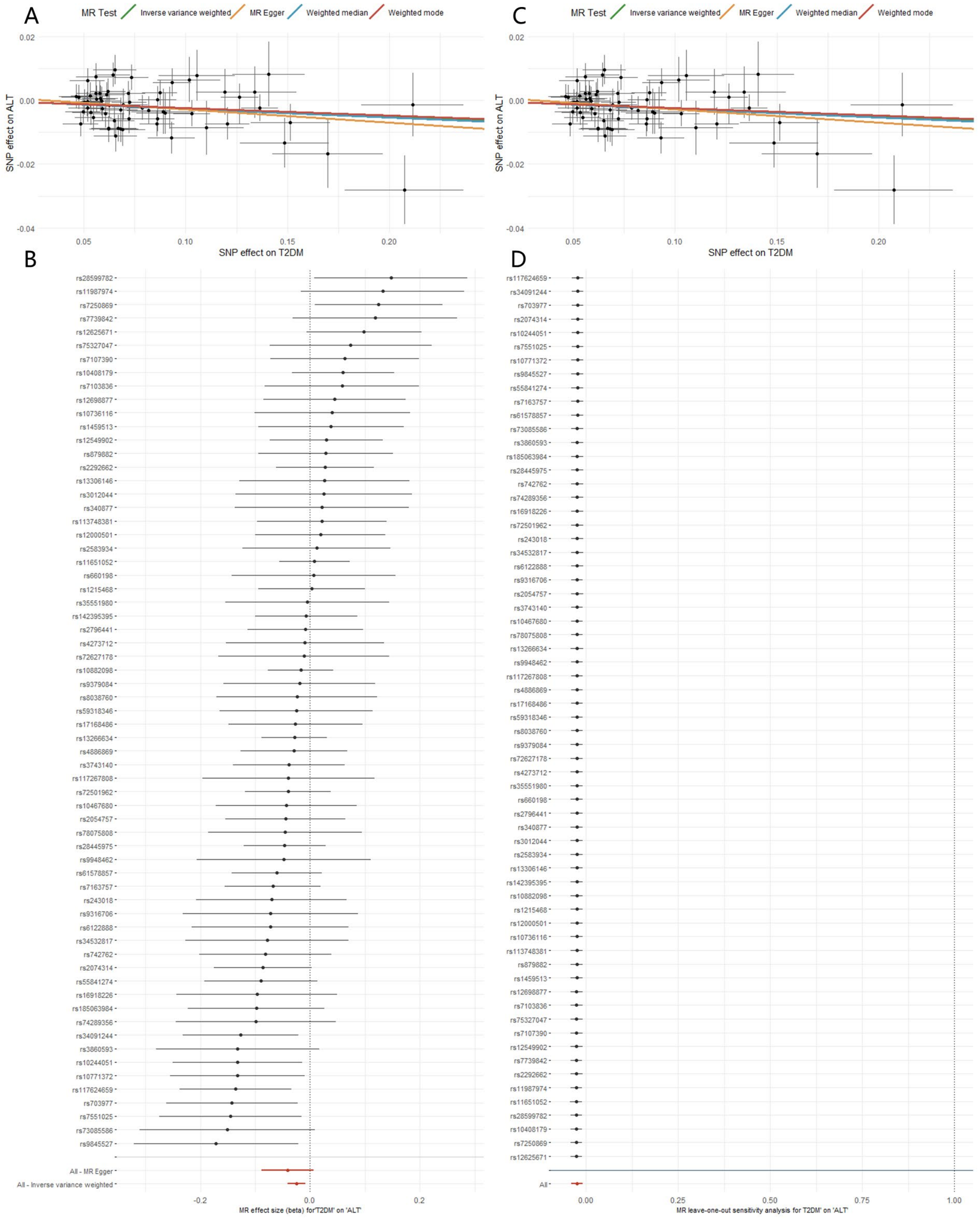

Figure S2 (A). Scatter plot referring to the effect sizes of the **SNP-T2DM association (x-axis, log OR) and the SNP-ALT in East Asians** associations (y-axis, log OR) with standard error bars. The slopes of the lines correspond to causal estimates using every of the four special methods (IVW random effects, MR-Egger, weighted median and weighted mode).(B).The forest plot shows the estimate of the impact of genetically increased T2DM risk on ALT risk in East Asians, where each black point represents the log OR for ALT per standard deviation extend in log OR for T2DM, and red points showing the combined causal estimate using all SNPs together in a single instrument, using every of the two different methods (IVW and MR-Egger). Horizontal lines denote 95% confidence intervals. (C). Funnel plot displaying the relationship between the causal effect of T2DM on ALT in East Asians estimated using each individual SNP as a separate instrument against the inverse of the standard error of the causal estimate. Vertical lines exhibit the causal estimates using all SNPs blended into a single instrument for every of the two different methods (IVW random effects and MR-Egger). There is no tremendous asymmetry in the plot. (D). MR leave-one-out sensitivity analysis for T2DM on ALT in East Asians. Each black point represents the IVW MR method applied to estimate the causal impact of T2DM on ALT aside from that particular variant from the analysis. The red point depicts the IVW estimate using all SNPs. There are no instances where the exclusion of one unique SNP leads to dramatic adjustments in the standard result.

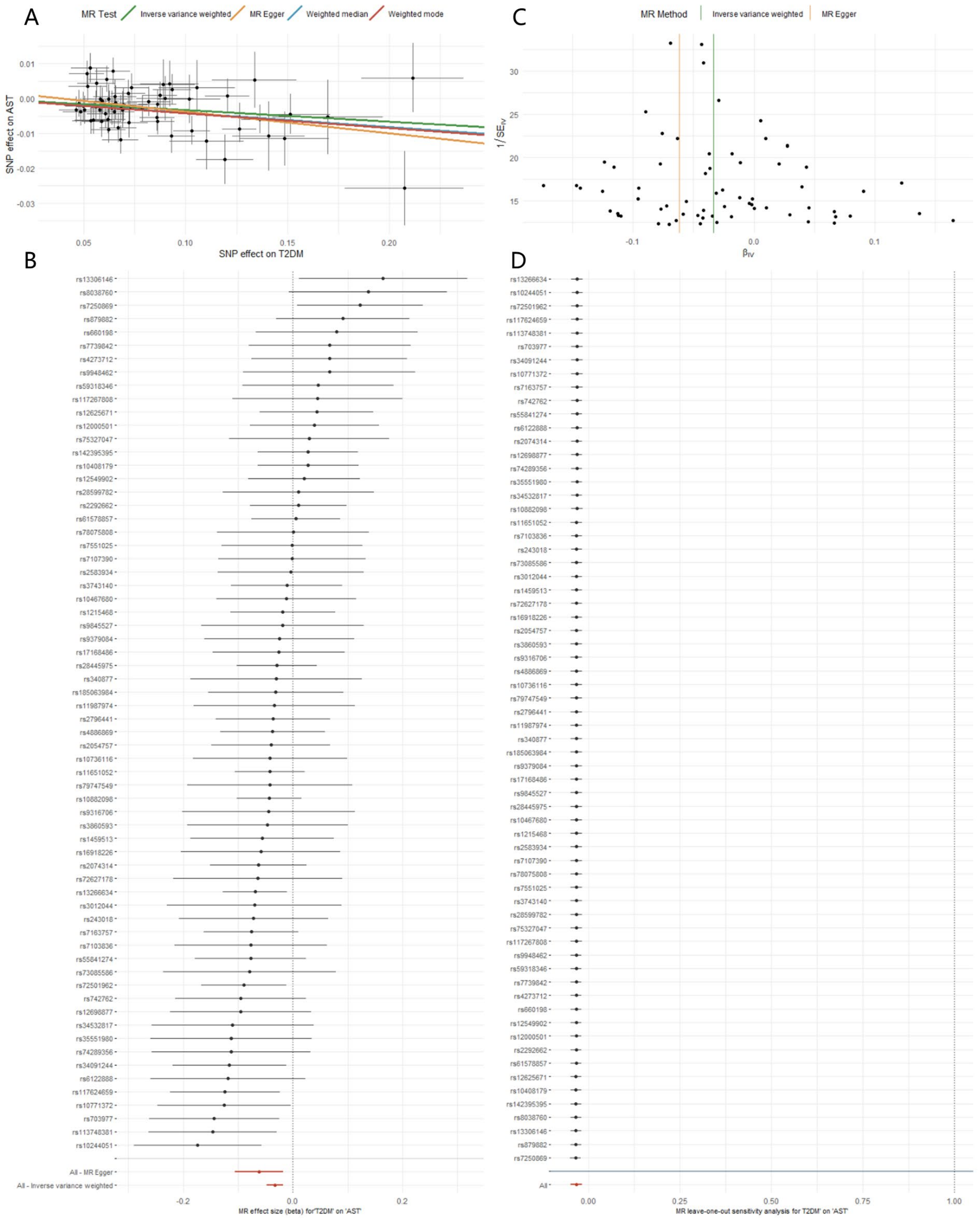

Figure S3 (A). Scatter plot referring to the effect sizes of the **SNP-T2DM association (x-axis, log OR) and the SNP-AST in East Asians** associations (y-axis, log OR) with standard error bars. The slopes of the lines correspond to causal estimates using every of the four special methods (IVW random effects, MR-Egger, weighted median and weighted mode). (B). Forest plot shows the estimate of the impact of genetically increased T2DM risk on AST risk in East Asians, where each black point represents the log OR for AST per standard deviation extend in log OR for T2DM, and red points showing the combined causal estimate using all SNPs together in a single instrument, using every of the two different methods (IVW random effects and MR-Egger). Horizontal lines denote 95% confidence intervals. (C). Funnel plot displaying the

relationship between the causal effect of T2DM on AST in East Asians estimated using each individual SNP as a separate instrument against the inverse of the standard error of the causal estimate. Vertical lines exhibit the causal estimates using all SNPs blended into a single instrument for every of the two different methods (IVW random effects and MR-Egger). There is no tremendous asymmetry in the plot. (D). MR leave-one-out sensitivity analysis for T2DM on AST in East Asians. Each black point represents the IVW MR method applied to estimate the causal impact of T2DM on AST aside from that particular variant from the analysis. The red point depicts the IVW estimate using all SNPs. There are no instances where the exclusion of one unique SNP leads to dramatic adjustments in the standard result.

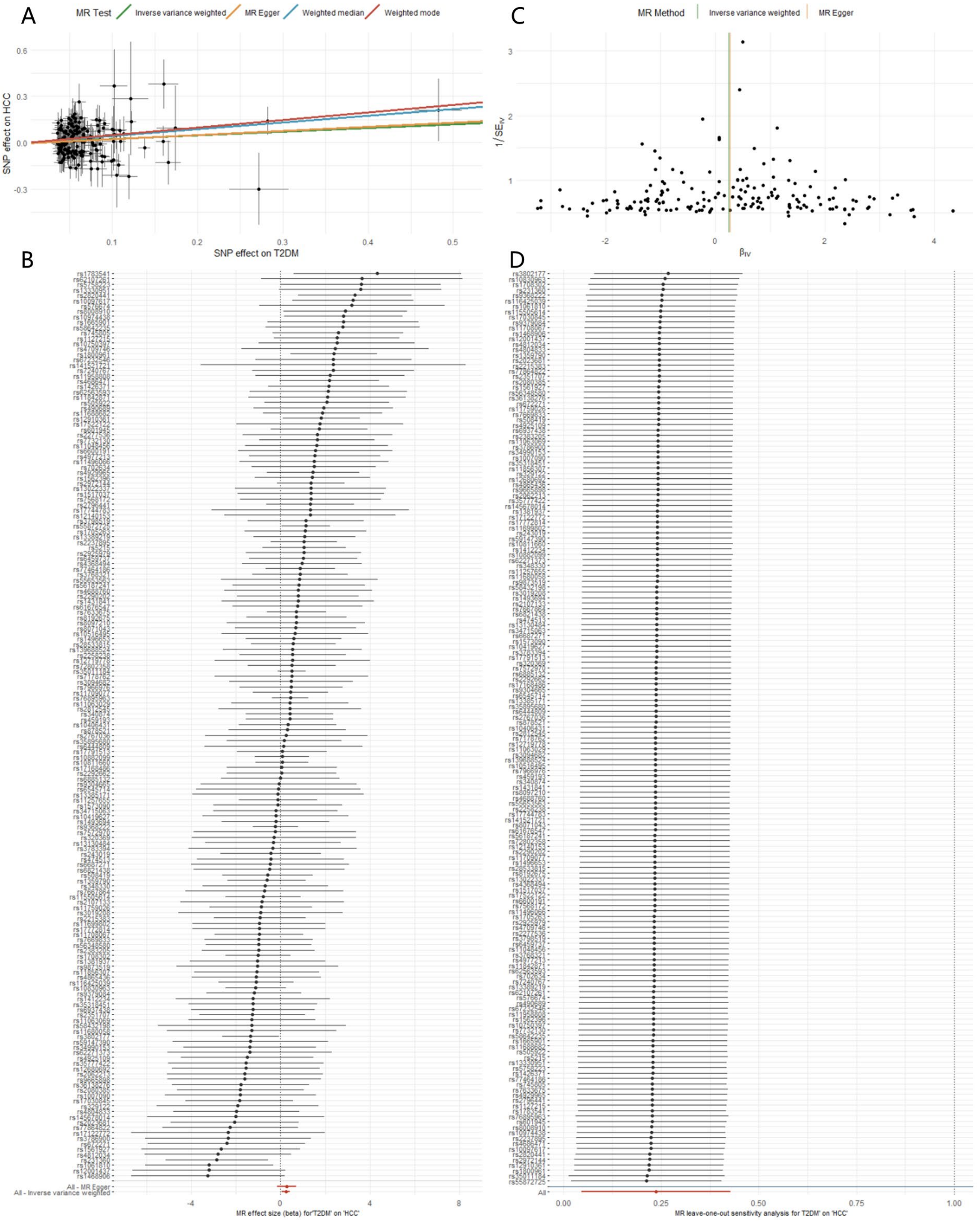

Figure S4 (A). Scatter plot referring to the effect sizes of the **SNP-T2DM association (x-axis, log OR) and the SNP-HCC in Europeans** associations (y-axis, log OR) with standard error bars. The slopes of the lines correspond to causal estimates using every of the four special methods (IVW random effects, MR-Egger, weighted median and weighted mode).(B).Forest plot shows the estimate of the impact of genetically increased T2DM risk on HCC risk in Europeans, where each black point represents the log OR for HCC per standard deviation extend in log OR for T2DM, and red points showing the combined causal estimate using all SNPs together in a single instrument, using every of the two different methods (IVW random effects and MR-Egger). Horizontal lines denote 95% confidence intervals. (C).Funnel plot displaying the relationship between the causal effect of T2DM on HCC in Europeans estimated using each individual SNP as a separate instrument against the inverse of the standard error of the causal estimate. Vertical lines exhibit the causal estimates using all SNPs blended into a single instrument for every of the two different methods (IVW random effects and MR-Egger). There is no tremendous asymmetry in the plot. (D). MR leave-one-out sensitivity analysis for HCC on NAFLD in Europeans. Each black point represents the IVW MR method applied to estimate the causal impact of T2DM on HCC aside from that particular variant from the analysis. The red point depicts the IVW estimate using all SNPs. There are no instances where the exclusion of one unique SNP leads to dramatic adjustments in the standard result.

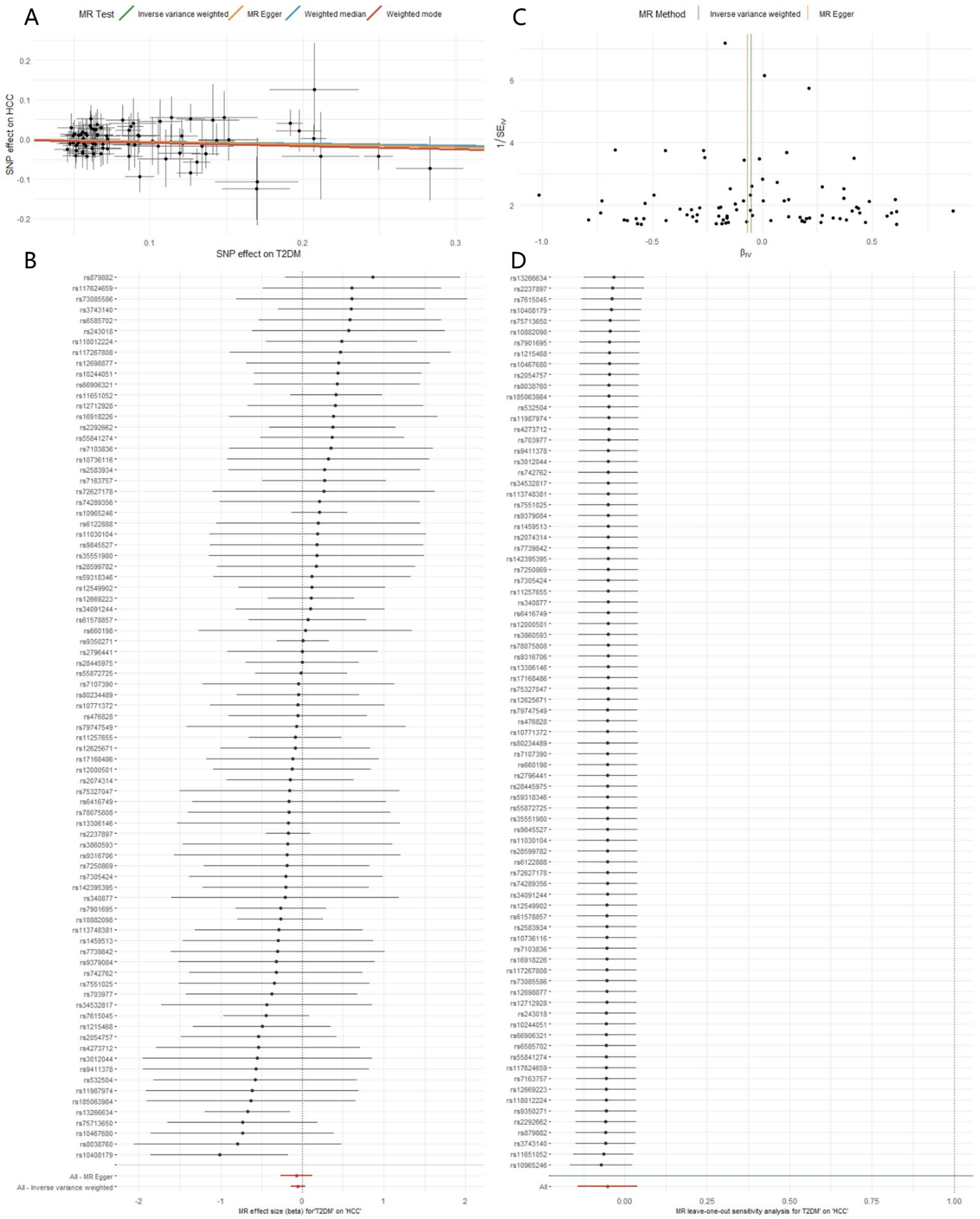

Figure S5 (A). Scatter plot referring to the effect sizes of the **SNP-T2DM association (x-axis, log OR) and the SNP-HCC in East Asians** associations (y-axis, log OR) with standard error bars. The slopes of the lines correspond to causal estimates using every of the four special methods (IVW random effects, MR-Egger, weighted median and weighted mode). (B). Forest plot shows the estimate of the impact of genetically increased T2DM risk on HCC risk in East Asians, where each black point represents the log OR for HCC per standard deviation extend in log OR for T2DM, and red points showing the combined causal estimate using all SNPs together in a single instrument, using every of the two different methods (IVW random effects and MR-Egger). Horizontal lines denote 95% confidence intervals. (C). Funnel plot displaying the

relationship between the causal effect of T2DM on HCC in East Asians estimated using each individual SNP as a separate instrument against the inverse of the standard error of the causal estimate. Vertical lines exhibit the causal estimates using all SNPs blended into a single instrument for every of the two different methods (IVW random effects and MR-Egger). There is no tremendous asymmetry in the plot. (D). MR leave-one-out sensitivity analysis for T2DM on HCC in East Asians. Each black point represents the IVW MR method applied to estimate the causal impact of T2DM on HCC aside from that particular variant from the analysis. The red point depicts the IVW estimate using all SNPs. There are no instances where the exclusion of one unique SNP leads to dramatic adjustments in the standard result.

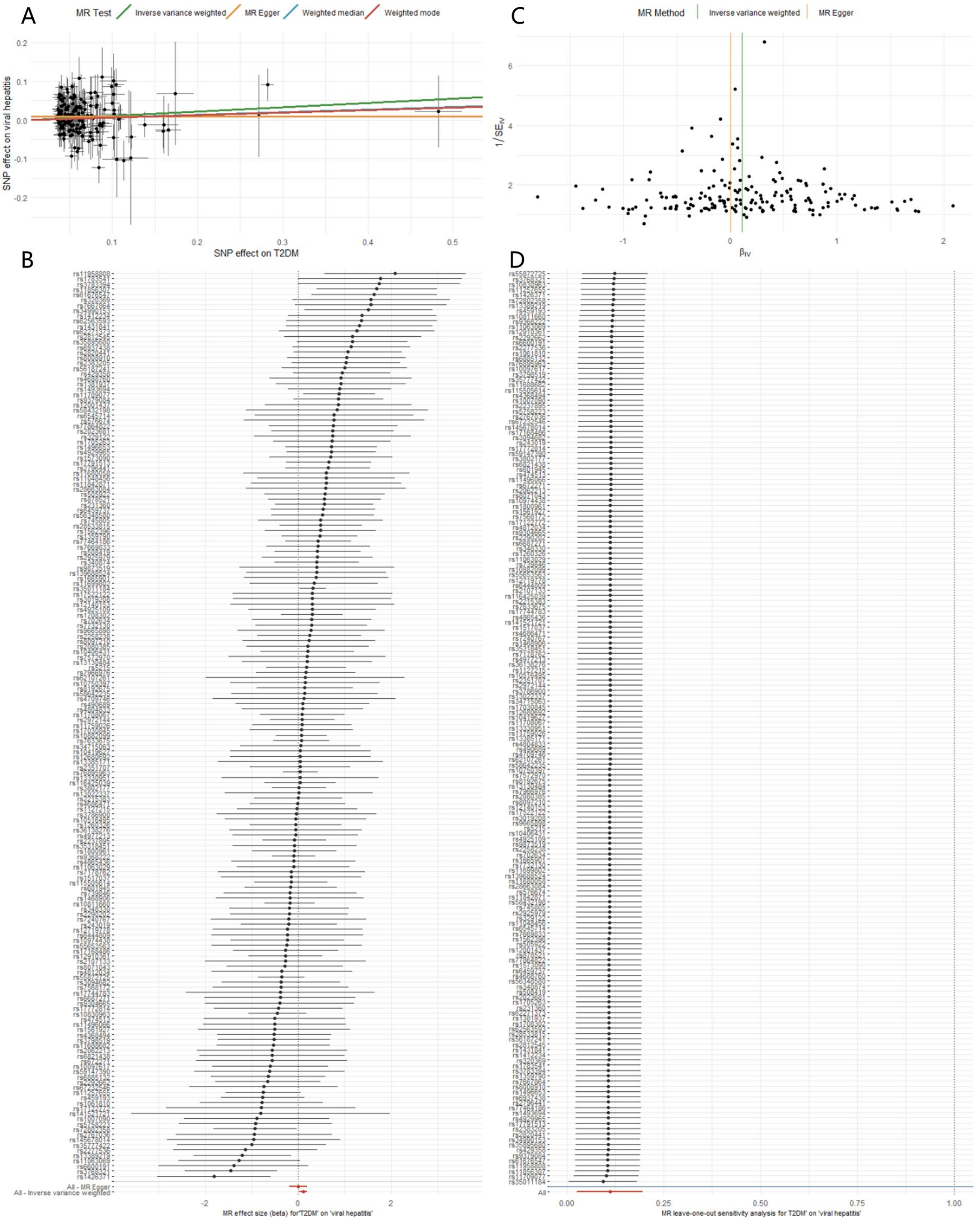

Figure S6 (A). Scatter plot referring to the effect sizes of the **SNP-T2DM association (x-axis, log OR) and the SNP-viral hepatitis in Europeans** associations (y-axis, log OR) with standard error bars. The slopes of the lines correspond to causal estimates using every of the four special methods (IVW random effects, MR-Egger, weighted median and weighted mode). (B). Forest plot shows the estimate of the impact of genetically increased T2DM risk on viral hepatitis risk in Europeans, where each black point represents the log OR for viral hepatitis per standard deviation extend in log OR for T2DM, and red points showing the combined causal estimate using all SNPs together in a single instrument, using every of the two different methods (IVW random effects and MR-Egger). Horizontal lines denote 95% confidence intervals. (C). Funnel plot displaying the relationship between the causal effect of T2DM on viral hepatitis in Europeans estimated using each individual SNP as a separate instrument against the inverse of the standard error of the causal estimate. Vertical lines exhibit the causal estimates using all SNPs blended into a single instrument for every of the two different methods (IVW random effects and MR-Egger). There is no tremendous asymmetry in the plot. (D). MR leave-one-out sensitivity analysis for viral hepatitis on NAFLD in Europeans. Each black point represents the IVW MR method applied to estimate the causal impact of T2DM on viral hepatitis aside from that particular variant from the analysis. The red point depicts the IVW estimate using all SNPs. There are no instances where the exclusion of one unique SNP leads to dramatic adjustments in the standard result.

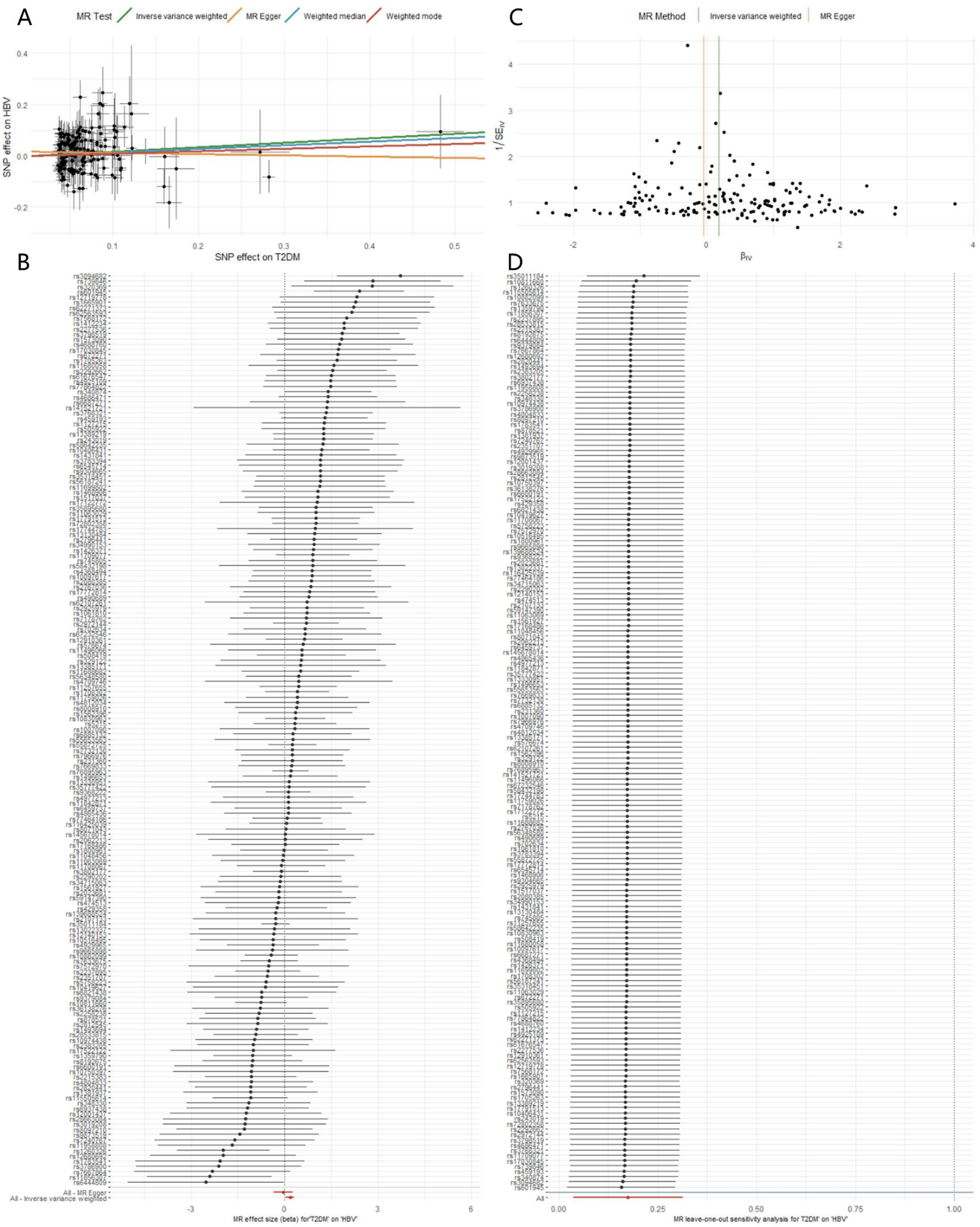

Figure S7 (A). Scatter plot referring to the effect sizes of the **SNP-T2DM association (x-axis, log OR) and the SNP-HBV in Europeans** associations (y-axis, log OR) with standard error bars. The slopes of the lines correspond to causal estimates using every of the four special methods (IVW random effects, MR-Egger, weighted median and weighted mode).(B).Forest plot shows the estimate of the impact of genetically increased T2DM risk on HBV risk in Europeans, where each black point represents the log OR for HBV per standard deviation extend in log OR for T2DM, and red points showing the combined causal estimate using all SNPs together in a single instrument, using every of the two different methods (IVW random effects and MR-Egger). Horizontal lines denote 95% confidence intervals. (C). Funnel plot displaying

the relationship between the causal effect of T2DM on HBV in Europeans estimated using each individual SNP as a separate instrument against the inverse of the standard error of the causal estimate. Vertical lines exhibit the causal estimates using all SNPs blended into a single instrument for every of the two different methods (IVW random effects and MR-Egger). There is no tremendous asymmetry in the plot. (D). MR leave-one-out sensitivity analysis for T2DM on HBV in Europeans. Each black point represents the IVW MR method applied to estimate the causal impact of T2DM on HBV aside from that particular variant from the analysis. The red point depicts the IVW estimate using all SNPs. There are no instances where the exclusion of one unique SNP leads to dramatic adjustments in the standard result.

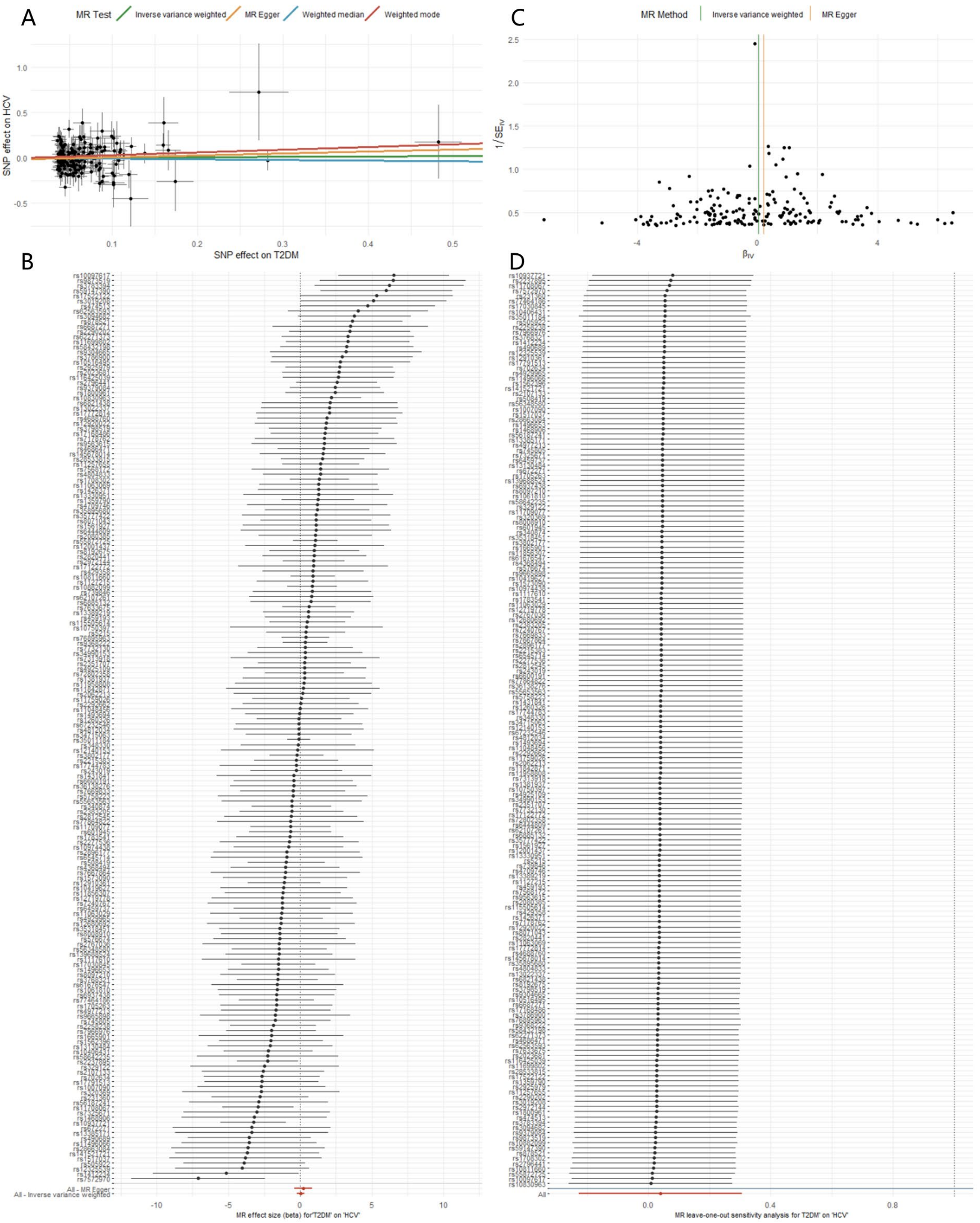

Figure S8 (A). Scatter plot referring to the effect sizes of the **SNP-T2DM association (x-axis, log OR) and the SNP-HCV in Europeans** associations (y-axis, log OR) with standard error bars. The slopes of the lines correspond to causal estimates using every of the four special methods (IVW random effects, MR-Egger, weighted median and weighted mode). (B). Forest plot shows the estimate of the impact of genetically increased T2DM risk on HCV risk in Europeans, where each black point represents the log OR for HCV per standard deviation extend in log OR for T2DM, and red points showing the combined causal estimate using all SNPs together in a single instrument, using every of the two different methods (IVW random effects and MR-Egger). Horizontal lines denote 95% confidence intervals. (C). Funnel plot displaying the relationship between the causal effect of T2DM on HCV in Europeans estimated using each individual SNP as a separate instrument against the inverse of the standard error of the causal estimate. Vertical lines exhibit the causal estimates using all SNPs blended into a single instrument for every of the two different methods (IVW random effects and MR-Egger). There is no tremendous asymmetry in the plot. (D). MR leave-one-out sensitivity analysis for T2DM on HCV in Europeans. Each black point represents the IVW MR method applied to estimate the causal impact of T2DM on HCV aside from that particular variant from the analysis. The red point depicts the IVW estimate using all SNPs. There are no instances where the exclusion of one unique SNP leads to dramatic adjustments in the standard result.

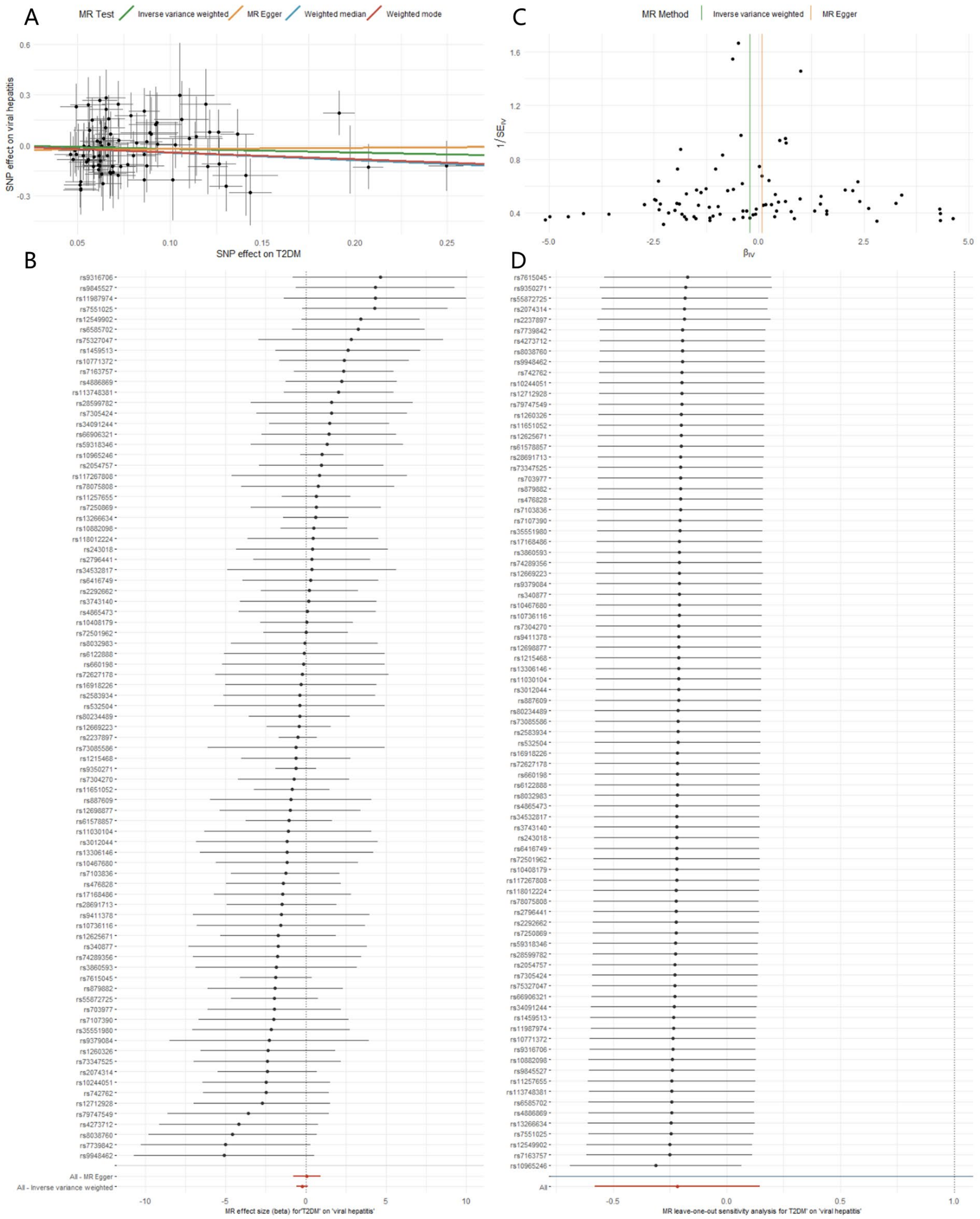

Figure S9 (A). Scatter plot referring to the effect sizes of the **SNP-T2DM association (x-axis, log OR) and the SNP-viral hepatitis in East Asians** associations (y-axis, log OR) with standard error bars. The slopes of the lines correspond to causal estimates using every of the four special methods (IVW random effects, MR-Egger, weighted median and weighted mode).(B).Forest plot shows the estimate of the impact of genetically increased T2DM risk on viral hepatitis risk in East Asians, where each black point represents the log OR for viral hepatitis per standard deviation extend in log OR for T2DM, and red points showing the combined causal estimate using all SNPs together in a single instrument, using every of the two different methods (IVW random effects and MR-Egger). Horizontal lines denote 95% confidence intervals. (C). Funnel

plot displaying the relationship between the causal effect of T2DM on viral hepatitis in East Asians estimated using each individual SNP as a separate instrument against the inverse of the standard error of the causal estimate. Vertical lines exhibit the causal estimates using all SNPs blended into a single instrument for every of the two different methods (IVW random effects and MR-Egger). There is no tremendous asymmetry in the plot. (D). MR leave-one-out sensitivity analysis for viral hepatitis on NAFLD in East Asians. Each black point represents the IVW MR method applied to estimate the causal impact of T2DM on viral hepatitis aside from that particular variant from the analysis. The red point depicts the IVW estimate using all SNPs. There are no instances where the exclusion of one unique SNP leads to dramatic adjustments in the standard result.

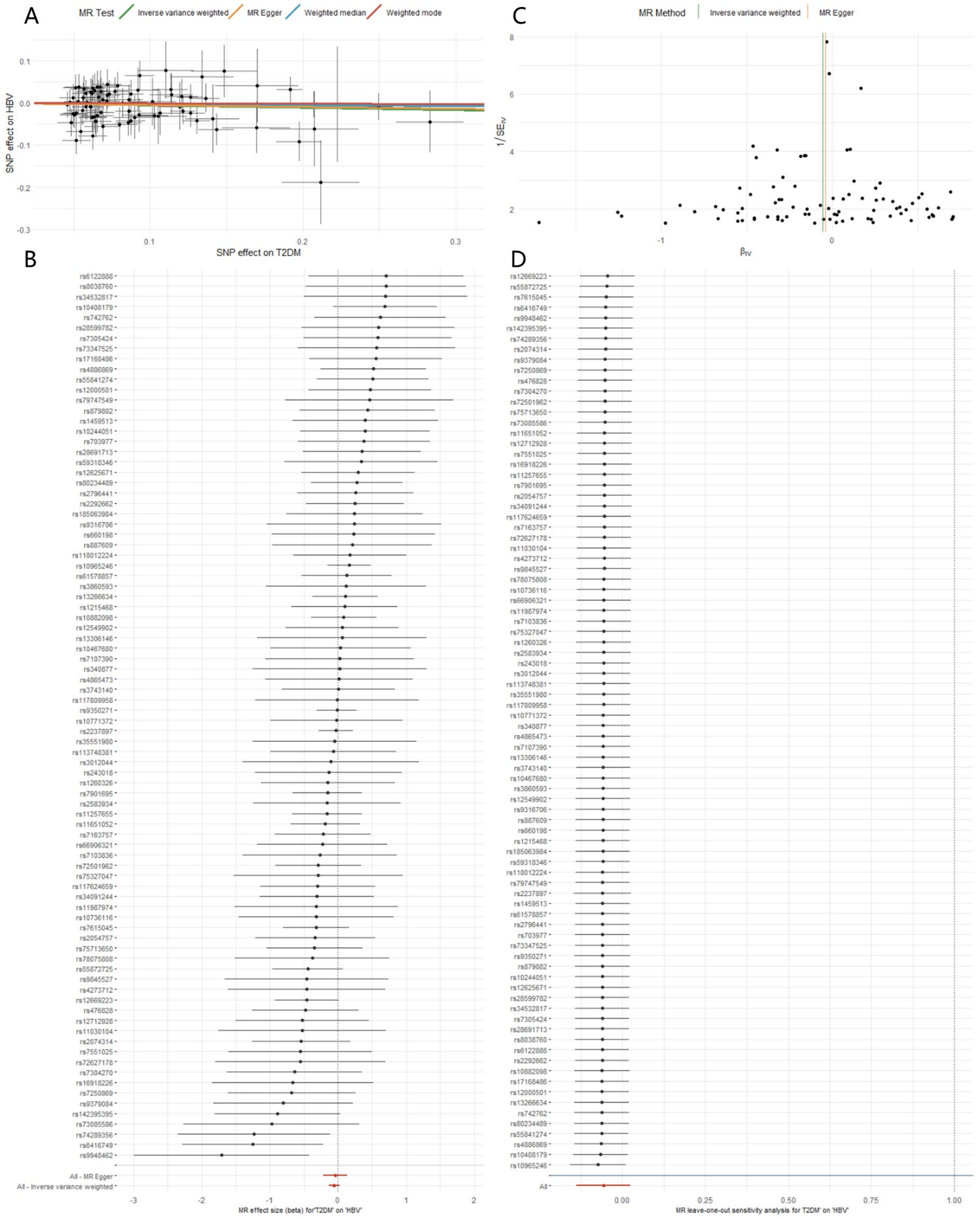

Figure S10 (A). Scatter plot referring to the effect sizes of the **SNP-T2DM association (x-axis, log OR) and the SNP-HBV in East Asians** associations (y-axis, log OR) with standard error bars. The slopes of the lines correspond to causal estimates using every of the four special methods (IVW random effects,MR-Egger,weighted median and weighted mode).(B).Forest plot shows the estimate of the impact of genetically increased T2DM risk on HBV risk in East Asians, where each black point represents the log OR for HBV per standard deviation extend in log OR for T2DM, and red points showing the combined causal estimate using all SNPs together in a single instrument, using every of the two different methods (IVW random effects and MR-Egger). Horizontal lines denote 95% confidence intervals. (C). Funnel plot displaying the relationship between the causal effect of T2DM on HBV in East Asians estimated using each individual SNP as a separate instrument against the inverse of the standard error of the causal estimate. Vertical lines exhibit the causal estimates using all SNPs blended into a single instrument for every of the two different methods (IVW random effects and MR-Egger). There is no tremendous asymmetry in the plot. (D). MR leave-one-out sensitivity analysis for T2DM on HBV in East Asians. Each black point represents the IVW MR method applied to estimate the causal impact of T2DM on HBV aside from that particular variant from the analysis. The red point depicts the IVW estimate using all SNPs. There are no instances where the exclusion of one unique SNP leads to dramatic adjustments in the standard result.

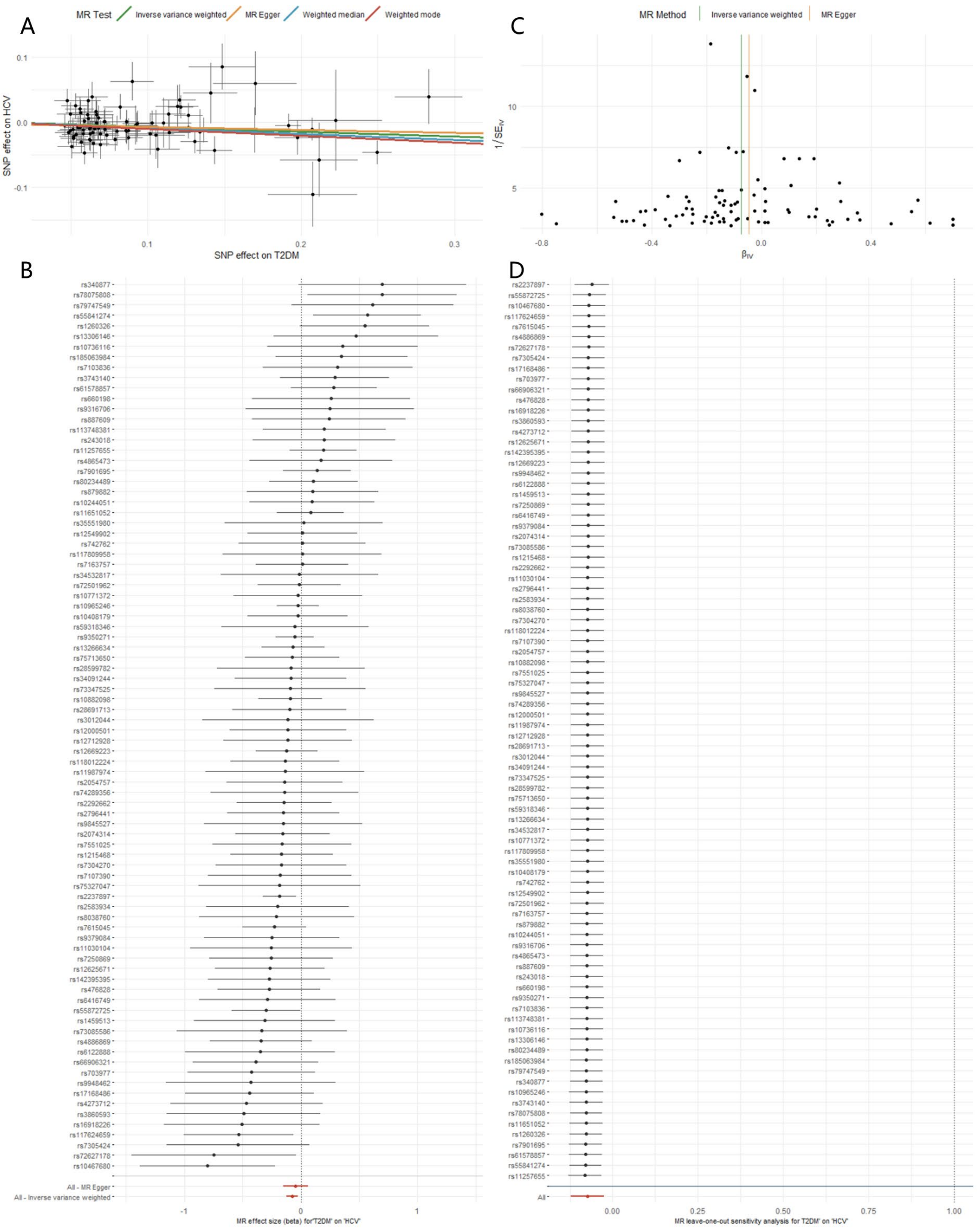

Figure S11 (A). Scatter plot referring to the effect sizes of the **SNP-T2DM association (x-axis, log OR) and the SNP-HCV in East Asians** associations (y-axis, log OR) with standard error bars. The slopes of the lines correspond to causal estimates using every of the four special methods (IVW random effects, MR-Egger, weighted median and weighted mode).(B).Forest plot shows the estimate of the impact of genetically increased T2DM risk on HCV risk in East Asians, where each black point represents the log OR for HCV per standard deviation extend in log OR for T2DM, and red points showing the combined causal estimate using all SNPs together in a single instrument, using every of the two different methods (IVW random effects and MR-Egger). Horizontal lines denote 95% confidence intervals. (C). Funnel plot displaying the

relationship between the causal effect of T2DM on HCV in East Asians estimated using each individual SNP as a separate instrument against the inverse of the standard error of the causal estimate. Vertical lines exhibit the causal estimates using all SNPs blended into a single instrument for every of the two different methods (IVW random effects and MR-Egger). There is no tremendous asymmetry in the plot. (D). MR leave-one-out sensitivity analysis for T2DM on HCV in East Asians. Each black point represents the IVW MR method applied to estimate the causal impact of T2DM on HCV aside from that particular variant from the analysis. The red point depicts the IVW estimate using all SNPs. There are no instances where the exclusion of one unique SNP leads to dramatic adjustments in the standard result.
